# Production and purification of polymerization-competent HIV-1 capsid protein p24 (CA) in NiCo21(DE3) *Escherichia coli*

**DOI:** 10.1186/1472-6750-13-107

**Published:** 2013-12-04

**Authors:** Sin Yeang Teow, Siti Aisyah Mualif, Tasyriq Che Omar, Chew Yik Wei, Narazah Mohd Yusoff, Syed A Ali

**Affiliations:** 1 Oncological and Radiological Sciences, Advanced Medical and Dental Institute, Universiti Sains Malaysia, Bertam, 13200 Kepala Batas, Pulau Pinang, Malaysia; 2 Regenerative Medicine, Advanced Medical and Dental Institute, Universiti Sains Malaysia, 13200 Kepala Batas, Pulau Pinang, Malaysia

## Abstract

**Background:**

HIV genome is packaged and organized in a conical capsid, which is made up of ~1,500 copies of the viral capsid protein p24 (CA). Being a primary structural component and due to its critical roles in both late and early stages of the HIV replication cycle, CA has attracted increased interest as a drug discovery target in recent years. Drug discovery studies require large amounts of highly pure and biologically active protein. It is therefore desirable to establish a simple and reproducible process for efficient production of HIV-1 CA.

**Result:**

In this work, 6-His-tagged wild type CA from HIV-1 (NL4.3) was expressed in rare tRNA-supplemented NiCo21(DE3) *Escherichia coli*, and its production was studied in shake flask culture condition of expression. Influences of various key cultivation parameters were examined to identify optimal conditions for HIV-1 CA production. It was found that a culture temperature of 22°C and induction with 0.05 mM IPTG at the early stage of growth were ideal, leading to a maximum biomass yield when grown in Super broth supplemented with 1% glucose. With optimized culture conditions, a final biomass concentration of ~27.7 g L^-1^ (based on optical density) was obtained in 12 hours post-induction, leading to a yield of about ~170 mg L^-1^ HIV-1 CA. A two-step purification strategy (chitin beads + IMAC) was employed, which efficiently removed metal affinity resin-binding bacterial proteins that contaminate recombinant His-tagged protein preparation, and resulted in highly pure HIV-1 CA. The purified protein was capable of polymerization when tested in an in vitro polymerization assay.

**Conclusions:**

By using this optimized expression and purification procedure, milligram amounts of highly pure and polymerization-competent recombinant HIV-1 CA can be produced at the lab-scale and thus used for further biochemical studies.

## Background

Human immunodeficiency virus (HIV) causes acquired immunodeficiency syndrome (AIDS), a progressive immune disorder that allows life-threatening opportunistic infections, cardiovascular diseases, and cancers to thrive. The capsid protein p24 (CA) plays seminal roles in both late and early stages of the HIV replication cycle [1]. HIV-1 CA is considered an important target for developing novel drugs to treat AIDS. For example, a small molecule, CAP-1, and two versions of a peptide inhibitor, CAI and NYAD-1, have been reported that target HIV-1 CA in vitro and interfere with its function in infected cells [2-4]. In another study, two small compounds PF-3450074 and PF-3759857 have shown to be active against HIV-1 in low μM concentration and latter (PF-3759857) against HIV-2 too [5]. More recently, compounds derived from benzodiazepines (BD) and the benzimidazoles (BM) series of chemicals have shown to prevent virion release and inhibited the formation of the mature capsid [6]. These studies require milligram quantities of the CA in soluble and active form. However, high commercial cost may limit its use in studies carried out at academic level.

The HIV-1 CA has been produced in bacterium *Escherichia coli*[7-11], yeast *Pichea pastoris*[12], plants [13-15], and baculovirus-insect cells [16]. However, existing methodologies rely on sequence modifications and several purification rounds involving precipitation and multiple chromatographic steps to obtain CA from host’ contaminating proteins.

Due to the presence of rare codons and low solubility of over-expressed recombinant protein, it has been challenging to obtain large quantities of HIV-1 CA. The aim of this study was to establish a convenient and relatively fast purification procedure for obtaining large amounts of biologically active HIV-1 CA at the laboratory scale. The expression and purification method described here is simple, expresses protein from the wild type *p*24 gene, and yields up to 170 mg of highly pure HIV-1 CA suitable for polymerization experiments.

## Methods

### Bacterial strains, plasmids, and media

The *E. coli* strains DH5α (NEB #C2987H) and NiCo21(DE3) (NEB #C2529H) were used for cloning and expression experiments respectively. Bacteria were aerobically grown in LB broth (10 g L^-1^ bacto-tryptone; 5 g L^-1^ yeast extract; 5 g L^-1^ NaCl; pH 7.0) or on LB agar (10 g L^-1^ bacto-tryptone; 5 g L^-1^ yeast extract; 5 g L^-1^ NaCl; 15 g L^-1^ Agar; pH 7.0) at various temperatures and in the absence or presence of Ampicillin (100 μg mL^-1^) and/or Chloramphenicol (25 μg mL^-1^). Bacterial strains were stored in LB broth plus 50% glycerol at -80°C. In some experiments, NiCo21(DE3) were transformed with rare tRNA supplementing plasmids pACYC-RIL (Stratagene), pRARE2 (Novagen), and pLysSRARE2 (Novagen). In some experiments, NiCo21(DE3) were grown in M9 broth (0.5 g L^-1^ NaCl; 1 g L^-1^ NH_4_Cl; 3 g L^-1^ KH_2_PO_4_; 6.78 g L^-1^ Na_2_HPO_4_.; 2 mM MgSO_4_; 0.1 mM CaCl_2_; 10 g L^-1^ glucose. pH 7.0), Super broth (32 g L^-1^ bacto-tryptone; 20 g L^-1^ yeast extract; 5 g L^-1^ NaCl; pH 7.0), or Terrific broth (12 g L^-1^ bacto-tryptone; 24 g L^-1^ yeast extract; 8 mL L^-1^ glycerol; 2.2 g L^-1^ KH_2_PO_4_; 9.4 g L^-1^ K_2_HPO_4_. pH 7.0).

### Construction of plasmid expressing HIV-1 CA (pSA-HP24-6His)

**Figure 1:**
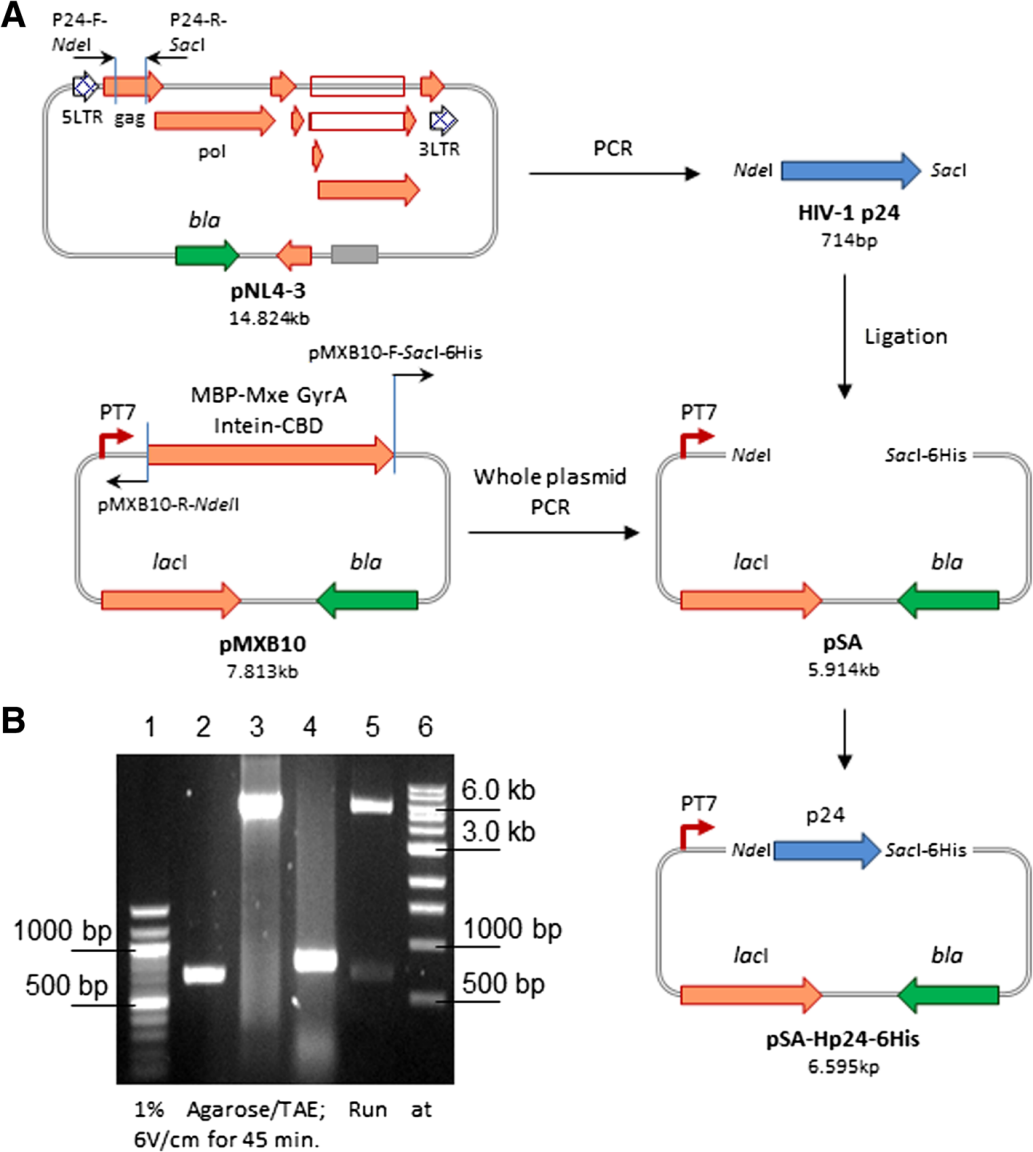
**Construction and verification of pSA-Hp24-6His vector.** We PCR amplified the p24 gene from pNL4.3 and cloned at *Nde*I/*Sac*I restriction sites in inversely PCRed pMXB10 vector **(A)**. Agarose gel electrophoresis analysis **(B)**. Lane 1, 100 bp DNA ladder (NEB#N0467S); Lane 2, PCR amplified 714 bp p24; Lane 3, inversely PCRed 5.91 kb pSA vector; Lane 4, representative colony PCR (806 bp); Lane 5, restriction analysis of pSA-Hp24-6His with *Nde*I/*Sac*I (5.89 kb vector backbone + 702 bp insert) ; Lane 6, 1 kb DNA ladder (NEB #N0468S).

Construction scheme of pSA-Hp24-6His is given in Figure 1A.

#### Preparation of insert (p24 with 5′ NdeI and 3′ SacI restriction enzyme sites)

The pNL4.3 plasmid (GenBank accession no. M19921) harboring complete genome of HIV-1 NL4.3 was obtained through the NIH AIDS Reagent Program and used as the template for PCR amplification of p24 gene encoding 231 residues of wild-type capsid protein p24. The p24 gene was PCR amplified using forward primer P24-F-*Nde*I (5′ GGTGGT*CATATG*CCTATAGTGCAGAACCTCCAG 3′, *Nde*I restriction site is in italics) and reverse primer P24-R-*Sac*I (5′ GGTGGT*GAGCTC*CAAAACTCTTGCTTTATGGCC 3′, *Sac*I restriction site is in italics) with high fidelity *Pfu* DNA polymerase (Thermo Scientific #EP0572) following manufacturer’s supplied protocol. The PCR product (714 bp amplicon) was purified using NucleoSpin® Gel and PCR Clean-up kit (MACHEREY-NAGEL GmbH & Co #740609) and restricted with *Nde*I (NEB # R0111L) and *Sac*I (NEB #R0156L) restriction enzymes following manufacturer’s supplied protocol.

#### Preparation of vector

Expression vector pMXB10 (NEB) was amplified by PCR using primers pMXB10-F-*Sac*I-6His (5′ CTC*GAGCTC*CACCATCACCATCACCATTGACTGCAGGAAGGGGATC 5′, *Sac*I restriction site is in italics) and pMXB10-R-*Nde*I (5′ GGCTCTTCC*CATATG*TATATCTCC 3′, *Nde*I restriction site is in italics) with *Pfu* polymerase. The PCRed plasmid (5.914 kb) was gel purified using NucleoSpin® Gel and PCR Clean-up kit and restricted with *Nde*I and *Sac*I restriction enzymes.

Insert and vector were ligated in a 1:3 molar ratio using T4 DNA ligase (NEB #M0202S), transformed in chemically competent DH5α *E. coli* cells, and selected on LB-agar plates (containing 100 ug mL^-1^ Ampicillin) after 18 h incubation at 30°C. A total of 10 randomly selected bacterial colonies were subjected to colony PCR using a vector-specific primer T7Up-F (5′ GATCCCGCGAAATTAATACG 3′) and an insert-specific primer P24-R (5′ GTGGAGCTCCAAAACTCTTGC 3′). Amplification of a 806 bp PCR product will be confirmatory for the successful cloning of P24 insert in pSA vector. Plasmid DNA was isolated from 3 colony PCR-positive clones and subjected to restriction analysis and DNA sequencing. We named this recombinant plasmid pSA-Hp24-6His.

### Expression of capsid protein p24 in pSA-HP24-6His-transformed NiCo21(DE3) E. coli

Sequencing-confirmed pSA-Hp24-6His was transformed into chemically competent NiCo21(DE3) *E coli* and transformants were selected on LB-Agar containing 100 μg mL^-1^ Ampicillin. For expression, a single colony from a freshly streaked (~18h) plate was inoculated in 10 mL of Ampicillin-supplemented LB broth. The starter culture was grown at 30°C while shaking at 250 rpm until the OD_600_ was approximately 1. The bacterial cells were centrifuged at 3000 × g for 10 min, re-suspend in fresh LB broth, and used to inoculate the main culture at 1:20 dilution (0.5 OD_600_). The culture was grown at 30°C while shaking at 250 rpm until the OD600 reached to 0.5-0.6. The cultures were then equilibrated to induction temperature (30, 22, or 18°C) and induced with various concentrations of Isopropylthio-β-galactoside (IPTG). Induced cultures were grown for different time lengths (6 hours at 30°C; 12 hours at 22°C; 16 hours at 18°C) and bacterial cells were pelleted by centrifugation at 5000 × g for 10 minutes in pre-weighed centrifuge tubes/bottles.

### Expression of HIV-1 CA in NiCo21(DE3) transformed with pACYC-RIL, pRARE2, and pLysSRARE2

The NiCo21(DE3) were individually transformed with pACYC-RIL, pRARE2, and pLysSRARE2 vectors and the transformants were selected on LB agar plates containing 25 μg mL^-1^ Chloramphenicol. Transformants were grown out and competent cells were prepared following Inoue’s protocol [17]. The pACYC-RIL, pRARE2, and pLysSRARE2-containing NiCo21(DE3) were then transformed with pSA-Hp24-6His vector and the transformants were selected on LB agar plates containing Ampicillin (100 μg mL^-1^) and Chloramphenicol (25 μg mL^-1^). Cultures were grown in presence of both the antibiotics and the expression protocol given above was essentially followed. To study the effect of various culture media on the expression of HIV-1 CA, cultures were grown in 1% glucose-supplemented M9 minimal broth, LB Broth, Terrific Broth, and Super Broth essentially following the expression protocol given above.

### Cell lysis and protein extraction

To every 1 gram of bacterial cell pellet, 4 mL of B-PER extraction reagent (Thermo Scientific #78248) supplemented with DNAse I (Thermo Scientific #90083) and protease cocktail (Thermo Scientific #87785) was added. The mixture was incubated at 22°C for 30 min on a nutator shaker. Soluble and insoluble proteins were partitioned by centrifuging bacterial cell lysate at 15,000 × g for 5–10 minutes at 4°C. Clear supernatant containing soluble proteins was passed through 0.45 μ membrane (Millipore #HPWP04700) and used for immobilized-metal affinity chromatography (IMAC). Insoluble lysed bacterial biomass was resuspended to original volume with phosphate buffered saline (PBS). Both, soluble and insoluble fractions were stored in small aliquots at -80°C for further analysis.

### Protein assays, SDS-PAGE, and immunoblotting

Total protein was quantitated using Qubit® Protein Assay Kit (Life Technologies – Invitrogen # Q33211) in a Qubit fluorometer (Life Technologies – Invitrogen). For SDS-PAGE, protein fractions were mixed with 5X reducing sample buffer (Thermo Scientific #39000), resolved on 12% (w/v) polyacrylamide gels, and detected with Coomassie blue G250 as described previously [18]. For immunoblot analysis, proteins were electroblotted onto the Hybond ECL nitrocellulose membrane (GE Healthcare Life Sceinces #RPN2020D). The membrane was washed in Tris-buffered saline (TBS) for 5 min, blocked with 5% nonfat milk in TTBS (TBS with 0.1% Tween 20) for 1 h by shaking at room temperature, processed for immunoblotting using either primary anti-His MAb (Thermo Scientific #MA1-21315) or anti-p24 MAb (NIH AIDS reagent program # 71–31) with shaking at 4°C overnight followed by a secondary HRP-conjugated IgG (H + L) antibody (Thermo Scientific # 32430). Protein bands were detected by SuperSignal West Pico Chemiluminescent substrate (Thermo Scientific # 34080) followed by image capture using FluorChem M Imager (ProteinSimple). Band intensities were determined using software AlphaView SA version 3.4.0. (ProteinSimple).

### Purification of HIV-1 CA

Recombinant HIV-1 CA was purified in two steps. First, the soluble protein fraction was pre-adsorbed on chitin resin (NEB #S6651) to get rid of bacterial Histidine-rich proteins. An appropriate amount of Chitin beads was added into a plastic chromatography column (BioRad #732-1010) and equilibrated in two resin-bed volumes of Equilibration/Wash Buffer (50 mM sodium phosphate, 500 mM sodium chloride, 10 mM imidazole; pH 7.4). Soluble protein fraction was diluted with an equal volume of Equilibration/Wash Buffer and added to the equilibrated chitin beads (1 ml of chitin resin for each volume of lysate corresponding to 1 gram of NiCo21(DE3) cell pellet). The column was then placed on an end-over-end rotator and revolved for 30 minutes at 4°C. Void volume containing target protein was eluted by gravity flow and used for IMAC.

For IMAC, HisPur™ Cobalt resin (Thermo Scientific #89965) was used following manufacturer’s protocol. Briefly, an appropriate amount of cobalt resin was added in 15 mL centrifuge tube and washed with two resin-bed volumes of Equilibration/Wash Buffer. The chitin bead pre-adsorbed soluble protein fraction was combined with equilibrated cobalt resin and mixed on an end-over-end rotator for 60 minutes at 4°C. The resin was then washed with Equilibration/Wash Buffer until the absorbance at 280 nm reached to the baseline. Bound protein was eluted using one resin-bed volume of Elution Buffer (50 mM sodium phosphate, 500 mM sodium chloride, 150 mM imidazole; pH 7.4). This step was repeated 2–3 times while saving individual fractions. Fractions were analyzed by SDS-PAGE/Western blot, dialyzed against phosphate buffered saline (PBS) using Slide-A-Lyzer Dialysis Cassettes, 7K MWCO (Thermo Scientific #66710) and stored at -80°C in small aliquots.

### Purification of HIV-1 CA on FPLC

The HIV-1 CA was purified using TALON® Superflow™ cobalt-based IMAC column on an FPLC system (AKTA purifier 900, GE). All buffers were prepared in deionized water; filtered through 0.2μm membrane, and degassed for 15 min. Column and all buffers were equilibrated to room temperature before use. The column was connected to the chromatography system by 'drop-to-drop’ method to avoid introducing air into the system. The column was equilibrated with 5 column volumes (CVs) of Binding buffer (50 mM sodium phosphate, 300 mM NaCl, pH 7.4). Sample was diluted 1:1 with Binding buffer and transferred into large-volume sample loop (Superloop 50 mL, GE) for sample loading. After sample loading, the column was washed with 10 CVs of Wash buffer (50 mM sodium phosphate, 300 mM NaCl, 5 mM imidazole, pH 7.4) until the absorbance reached a steady baseline. Protein was eluted in a linear imidazole gradient (5-150 mM in 50 mM sodium phosphate, 300 mM NaCl, pH 7.4) and collected in 1 mL fractions. The purified fractions were then analyzed by SDS-PAGE, dialyzed against phosphate buffered saline (PBS) using Slide-A-Lyzer Dialysis Cassettes, 7K MWCO (Thermo Scientific #66710), and stored at -80°C in small aliquots. The column was washed with 5 CVs of 20% ethanol and stored at 4°C.

### Polymerization assay for HIV-1 CA

A turbidometric assay was used to study *in vitro* polymerization of HIV-1 CA as described previously [19]. Final concentration of purified CA (40-80 μM) was mixed with 50 mM sodium phosphate buffer (pH8.0) in a total volume of 250 μL. The CA assembly was induced by adding 250 μL of 2.0 M NaCl (final concentration) in 50 mM sodium phosphate buffer, pH8.0. The mixture (500 μL) was briefly vortexed, immediately transferred to a silica cuvette (10 mm path length), and spectrophotometrically read at 350 nm wavelength at room temperature. The absorbance measurements were made every 10s for up to 60 min. The assembly rate was then set by plotting the absorbance versus time.

For polymerization inhibition, 50 μM purified CA was mixed with various concentrations (1, 5, 10 μM) of CA-specific anti-p24 antibody (NIH AIDS reagent program # 71–31) in 50 mM sodium phosphate buffer (pH8.0) in a total volume of 250 μL. The reaction was incubated at room temperature for 30 minutes. The CA assembly was induced by adding 250 μL of 2.0 M NaCl and polymerization monitored as described above. Anti-Acetylcholinesterase (AChE) antibody, clone AE-1 (Merck Millipore # MAB303) was used as negative control at a final concentration of 20 μM.

### Immunization and HIV-1 CA-specific antibody detection

Two 6-weeks old female BALB/c mice were individually primed by a single intradermal injection of 100 μg/100 μL CA in complete Freund’s Adjuvant (CFA). Mice were boosted with three successive injections of 100 μg/100 μL CA in Incomplete Freund’s Adjuvant (IFA) at an interval of two weeks. Serum was prepared from blood samples and serially diluted from 1:1000 to 1:100,000 and subjected to ELISA and used for immunoblot analysis.

### 293T cell culture and transfection

The 293T cells (ATCC #CRL-11268) were maintained in DMEM (Sigma #D5030) supplemented with 10%FBS (Life technologies # 16000–044) at 37°C in presence of 5% CO_2_. Cells (1×10^5^) were transfected with 0.5 μg of pNL4.3 (prepared using Endotoxin-free plasmid, Macherey Nagel # 740420) using 2 μL of X-tremeGene HP transfection reagent (Roche #06366236001) following supplied protocol. Cells were harvested 48 hours post-transfection, lysed with M-Per lysis reagent (Thermo Scientific #78503), and subjected to SDS-PAGE/Western blot analysis.

## Results

### Construction of plasmid expressing HIV-1 CA

To express HIV-1 capsid protein p24, we chose prokaryotic expression vector pMXB10 due to its ability of high level heterologous protein expression in BL21(DE3) *E. coli*. The 'ATG’ initiation codon is the part of *Nde*I restriction site and located at an optimal distance of 8 nucleotides from the ribosome binding site (RBS). We did not aim to express capsid protein p24 as a fusion protein with intein-CBD (for which the pMXB10 vector was originally designed) and therefore, we took out the entire 1.938 kb MBP-Mxe GyrA intein-CBD fragment by *PCR* amplification of the pMXB10. We also introduced a *Sac*I restriction site followed by 21 nucleotides encoding a 6His tag and a stop codon as shown in Figure 1A. Cloning of a 714 bp amplicon encoding 231 residues of wild-type HIV-1 capsid protein p24 at *Nde*I/*Sac*I sites resulted in a 6.595 kb recombinant plasmid pSA-Hp24-6His. We verified the pSA-Hp24-6His construct by restriction enzyme analysis (Figure 1B) and DNA sequencing (data not shown). The mRNA sequence encoding the capsid protein p24 is transcribed from an inducible T7 promoter and the resulting protein has a 6His tag at its carboxy-terminal to facilitate IMAC-mediated purification.

### Expression of HIV-1 CA in NiCo21(DE3) E. coli

**Figure 2:**
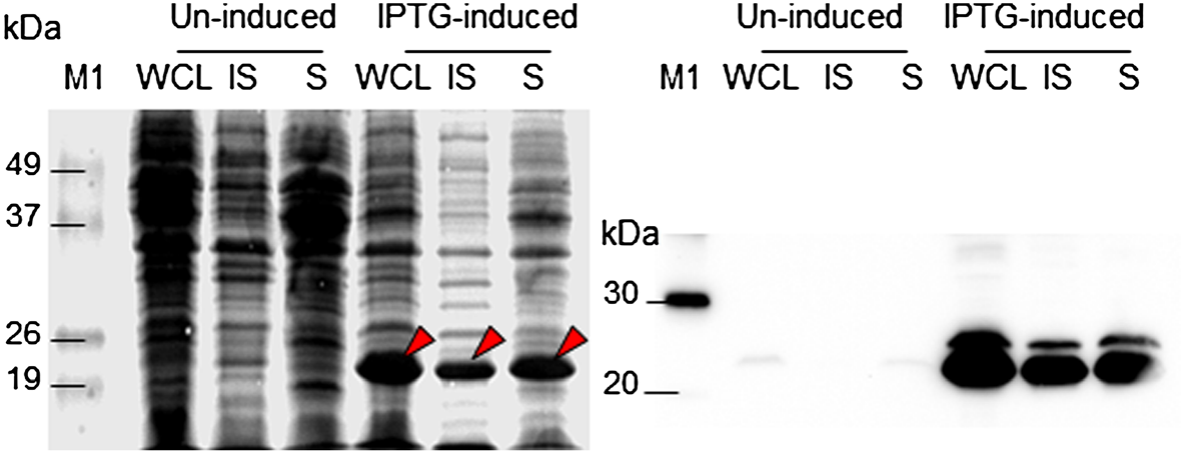
**SDS-PAGE/immunoblot analysis of recombinant HIV-1 CA.** NiCo21(DE3) *E. coli* were transformed with pSA-Hp24-6His vector and grown at 30°C for 6 hours, either induced with 0.4 mM IPTG, or un-induced. Cells were processed as described in Methods and labeled as whole cell lysate (WCL), insoluble fraction (IS), and soluble fraction (S). Eight microliter of each sample (WCL, IS, S) was mixed with 4X loading dye and electrophoresed on 12% gel. Proteins were transferred to nitrocellulose membrane and stained with MemCode™ Reversible Protein Stain Kit (Pierce) and photographed **(left panel)**. Membrane was then destained and subjected to immunoblot analysis using primary anti-p24 antibody and secondary anti-Mouse antibody. Membrane was developed using enhanced chemilumenscent reagent (Pierce) and image was captured **(right panel)**. Lane M1, BenchMark Pre-stained protein ladder; Lanes WCL, whole cell lysate; Lanes IS, insoluble fraction; Lanes S, soluble fraction; Lane M2, MagicMark XP Western Protein Standard.

We used NiCo21 (DE3) to express 6His-tagged HIV-1 CA using shake flask culture condition of expression. Plasmids containing retroviral sequences are instable in *E. coli*[20], especially when grown at 37°C. Therefore, we cultured the pSA-Hp24-6His-trandformed NiCo21(DE3) at 30°C until the OD_600_ reached to 0.5-0.6. We then induced the cultures with 0.4 mM IPTG and continued incubation at 30°C for another 6 hours while shaking. When subjected to SDS-PAGE analysis, the overexpressed CA appeared as approximately 24 kDa band in the IPTG-induced fractions (Figure 2, left panel). Overexpressed CA was present in both, insoluble (Figure 2, lane IS) and soluble fraction (Figure 2, lane S). Immunoblot analysis with anti-CA antibody confirmed that the overexpressed protein was HIV-1 CA (Figure 2, right panel). On the western blot, just above the CA band, a 25-26 kDA band was observed. Either it is a host protein that binds to CA or it is a product of transcriptional read-through. Since the anti-p24 antibody does not bind to the host proteins, we anticipate that this anti-p24 reactive 25-26 kDA band is a product of transcriptional read-through.

### Optimization of IPTG concentration and induction temperature

**Figure 3:**
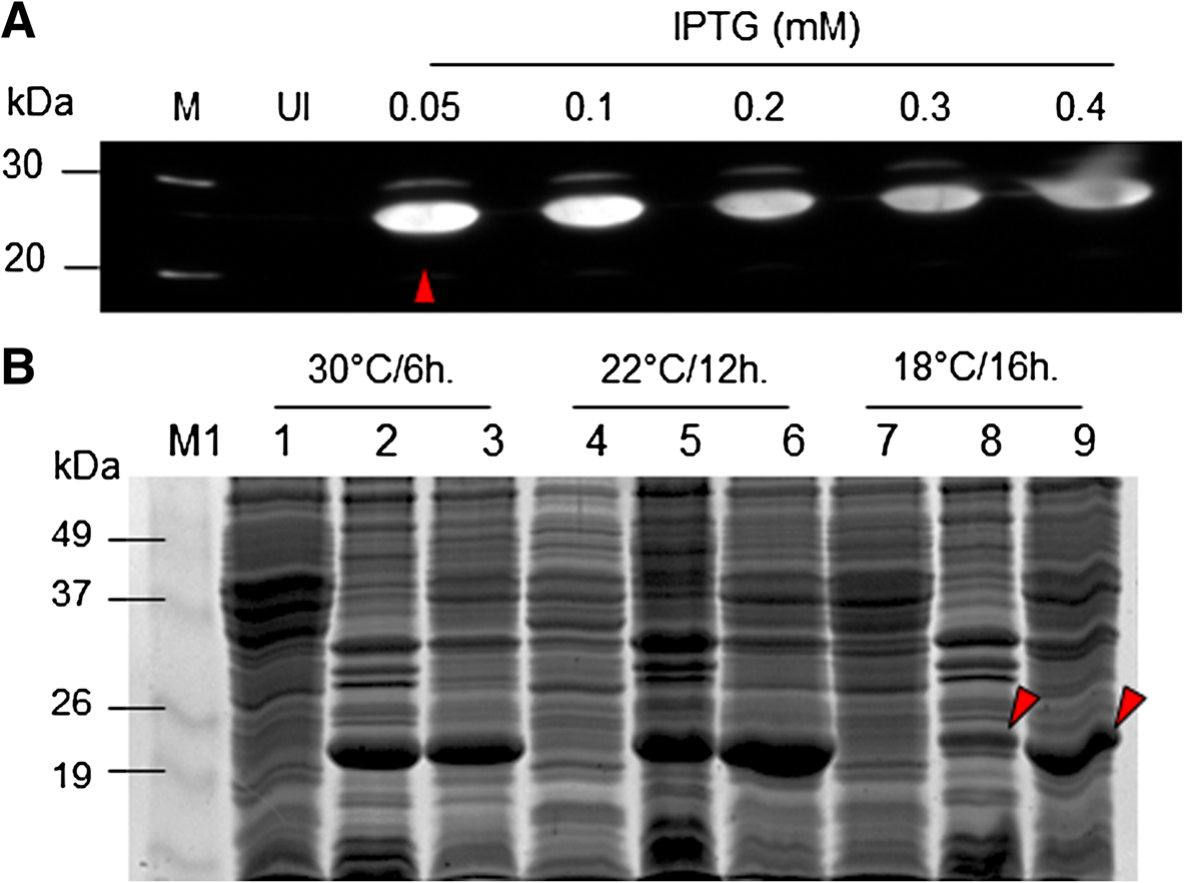
**Optimization of IPTG concentration and induction temperature for the expression of HIV-1 CA.***Determination of optimal inducer concentration:* The pSA-Hp24-6His-transformed NiCo21 (DE3) *E. col*i cultures (0.6OD_600_) were added with varying concentrations (0–0.4 mM) of IPTG and incubated at 30°C for 6 hours. Eight micro liter of cultures were mixed with 4X loading dye, boiled, and electrophoresed on 12% gels. Proteins were transferred to NC membranes and immunoblot analysis was carried out as described in Methods. **(A)**. *Determination of optimal induction temperature:* The pSA-Hp24-6His-transformed NiCo21(DE3) *E.coli* cultures (0.6OD_600_) were induced with 0.05 mM IPTG and incubated at 30, 22, and 18°C for 6, 12, and 18 hours respectively. Cultures were then processed to obtain insoluble (IS) and soluble (S) fractions and electrophoresed as described in Methods. Gels were stained with Coomassie Blue G250 and photographed **(B)**. Lane M1, BenchMark Pre-stained protein ladder; Lane 1: Un-induced culture at 30°C (soluble); Lane 2, IPTG-induced culture at 30°C (insoluble); Lane 3, IPTG-induced culture at 30°C (soluble); Lane 4: Un-induced culture at 22°C (soluble); Lane 5, IPTG-induced culture at 22°C (insoluble); Lane 6, IPTG-induced culture at 22°C (soluble); Lane 7: Un-induced culture at 18°C (soluble); Lane 8, IPTG-induced culture at 18°C (insoluble); Lane 9, IPTG-induced culture at 18°C (soluble).

Since induction with lower IPTG concentrations and/or at lower temperature may result in improved solubility of overexpressed protein [21], we tested various IPTG concentrations (0–0.4 mM) and induction temperatures (30, 22, and 18°C). Expression of HIV-1 CA was optimal at 0.05 mM of IPTG as determined by western blot analysis (Figure 3A). More overexpressed protein was present in soluble fraction compared to in-soluble fraction when cultures were induced with 0.05 mM IPTG and grown at 18°C for 16 hours (Figure 3B, lanes 8&9). However, larger amounts of CA were present in cultures induced with 0.05 mM IPTG and grown at 22°C for 12 hours (Figure 3B, lanes 5&6).

### Effect of rare tRNA supplementation on HIV-1 CA production in NiCo21(DE3) E. coli

**Figure 4:**
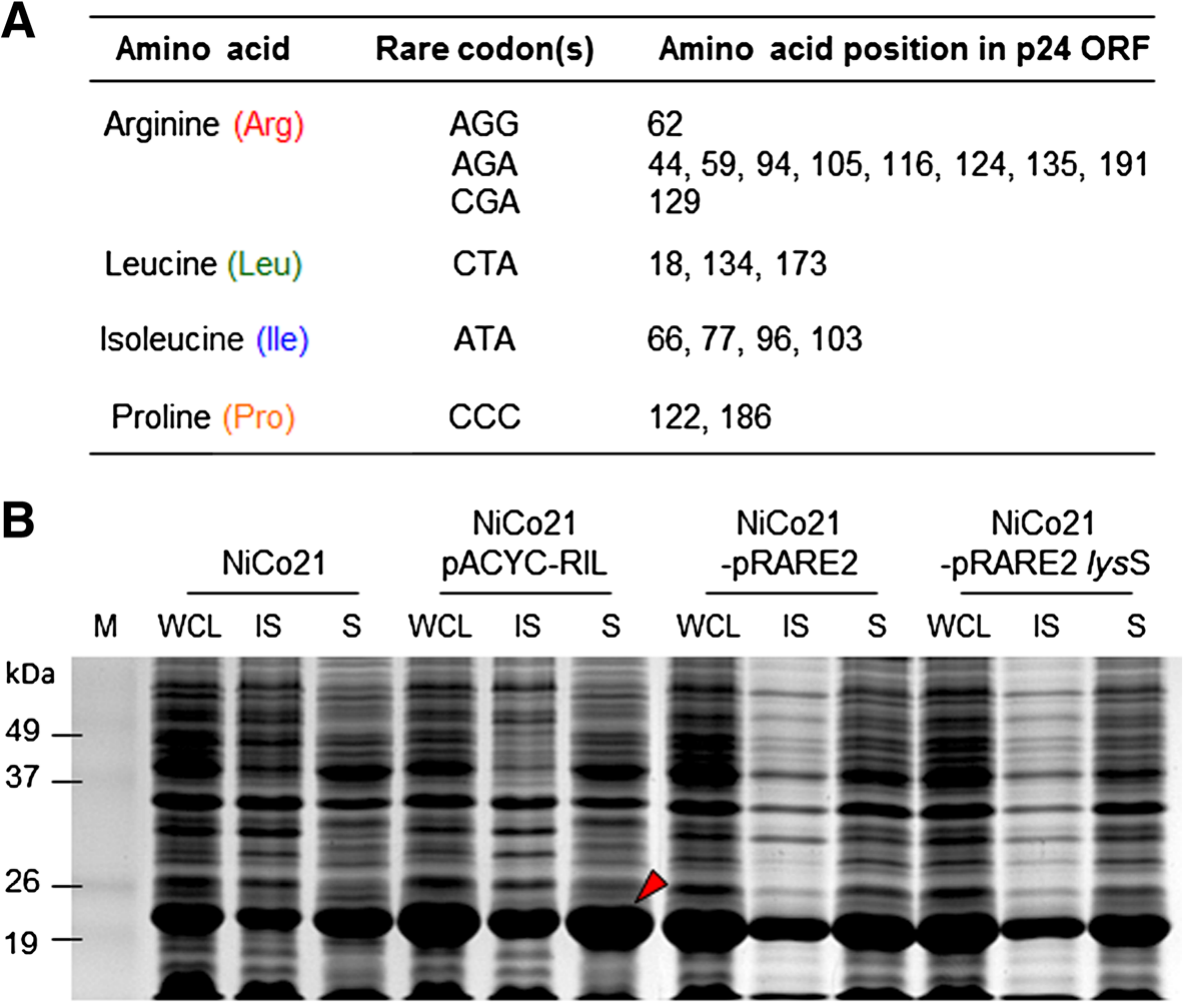
**Effect of rare tRNA supplementation on HIV-1 CA expression in NiCo21(DE3) *E. coli. ***Rare codon analysis of open reading frame (ORF) coding for HIV-1 p24 gene **(A)**. NiCo21(DE3) *E. coli* were transformed with pACYC-RIL or pRARE2 or pRARE2-lysS and subsequently transformed with pSA-Hp24-6His vectors and selected on LB + Cam + Amp plates. Cultures were grown in presence of Cam + Amp at 22°C for 12 hours, either induced with 0.05 mM IPTG, or un-induced. Cultures were processed to obtain whole cell lysate (WCL), insoluble (S), and soluble (S) fractions. Samples were electrophoresed on 12% gel, stained with Coomassie Blue G250, and photographed **(B)**. Lane M, BenchMark Pre-stained protein ladder; Lanes WCL, whole cell lysate; Lanes IS, insoluble fraction; Lanes S, soluble fraction.

Over-expression of recombinant proteins in *E. coli* may be significantly reduced or even stalled if the ORF that codes for the protein uses “rare” codons that are infrequently used by *E. coli*[21,22]. When subjected to 'rare’ codon analysis, 576 bp ORF coding for HIV-1 CA found to contain (a) three rare codons (AGG, AGA, CGA) for arginine at positions 44, 59, 62, 94, 105, 116, 124, 129, 135, 191; (b) one rare codon (CTA) for leucine at positions 18, 134, 173; (c) one rare codon (ATA) for isoleucine at positions 66, 77, 96, 103, 115; and (d) one rare codon (CCC) for proline at positions 122, 186 (Figure 4A). To find out whether CA expression could be improved by supplementing NiCo21(DE3) cells with plasmids expressing rare tRNA, we transformed NiCo21(DE3) with pACYC-RIL, pRARE2, and pLysSRARE2. The pACYC-RIL plasmid supplies tRNA for four rare codons (AUA, AGG, AGA, CUA), whereas pRARE2 for seven rare codons (AUA, AGG, AGA, CUA, CCC, CGG, and GGA). In addition to seven rare codons, transformation with pLysSRARE2 also results in lower background expression due to the presence of T7 lysozyme. When expressed in the presence of rare tRNA supplying plasmids, there was an improvement in CA expression (Figure 4B). However, overexpressed protein proportionally ended up in the insoluble fractions suggesting that bacterial protein folding machinery was saturated when CA was expressed in the presence of rare tRNA. We did not see a significant change in CA expression between the NiCo21(DE3) containing pACYC-RIL and pRARE2/pLysSRARE2. This suggests that supplementation with 4 rare codons was sufficient to improve CA expression. When compared side-by-side, we found NiCo21(DE3)/pACYC-RIL produced highest amount of CA (Figure 4B). Expression of CA in the presence or absence of rare tRNA had minimal effect on the growth of bacteria as determined by the total biomass yield (9.1 g L^-1^ for NiCo21(DE3) and 8.96 g L^-1^ for NiCo21(DE3) with pACYC-RIL). These experiments were repeated at 18°C but distribution of recombinant protein between soluble and insoluble fraction remained the same, and incubation at lower temperature did not result in more soluble CA (data not shown).

**Figure 5:**
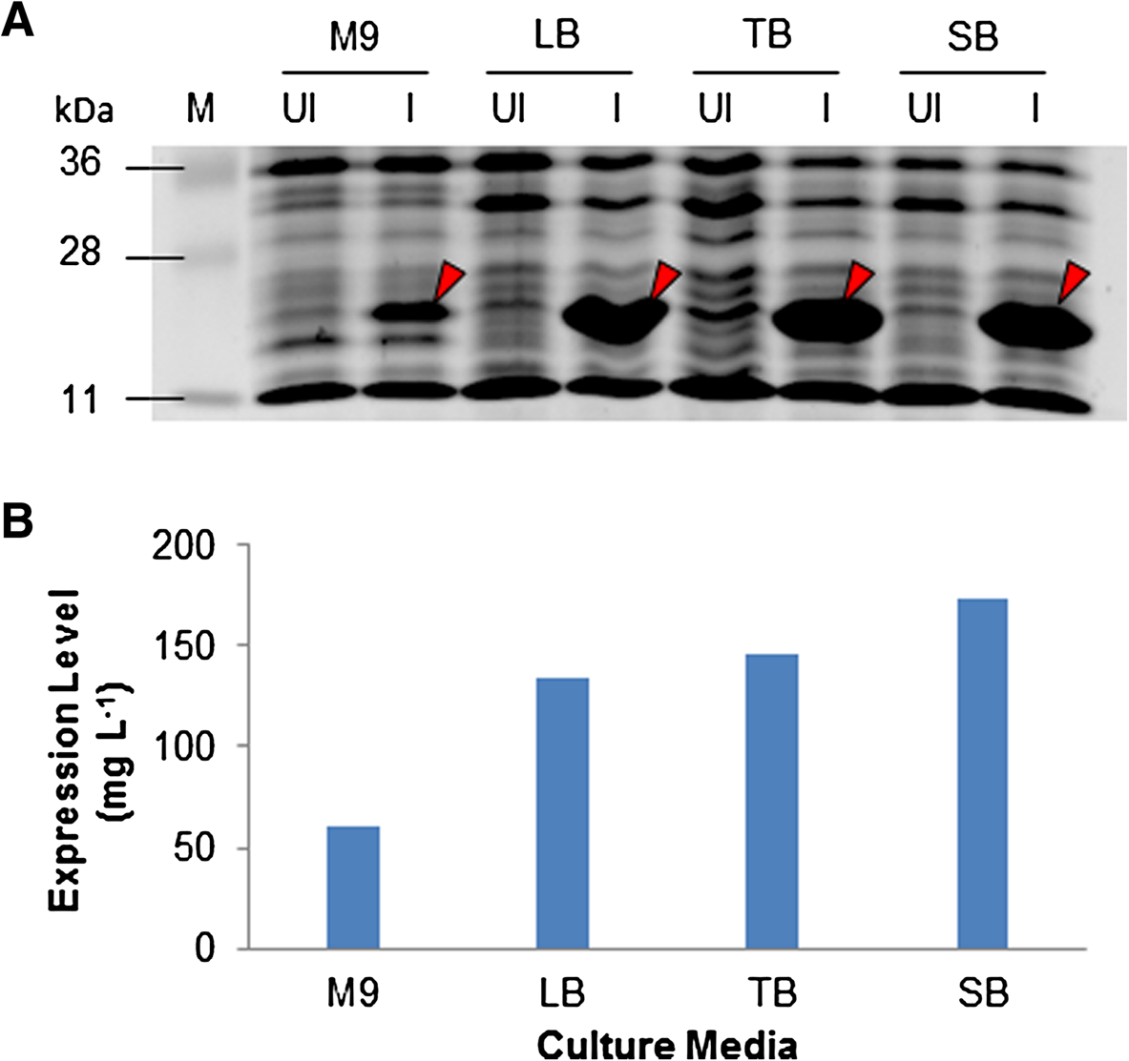
**Effect of cultivation medium composition on production of HIV-1 CA.** NiCo21(DE3)-pACYC-RIL expressing HIV-1 CA were grown in various cultivation media supplemented with 1% glucose and 0.05 mM IPTG at 22°C for 12 hours. Cultures were adjusted to 1.5OD_600_ (to normalize the biomass) and 8 uL of culture was analyzed using SDS-PAGE **(A)**. M, Pre-stained protein ladder; Lanes UI, un-induced; Lanes I, IPTG-induced. Comparison of HIV-1 CA production levels obtained using different growth media **(B)**. Cells were harvested, lysed, and subjected to Chitin/IMAC purification. Eluted protein was quantitated and HIV-1 CA produced from 1L of biomass was calculated.

We also studied the effect of cultivation medium composition both on the growth of the NiCo21(DE3)-pACYC-RIL and the production of the HIV-1 CA. Cells were grown in M9, LB, Super, and Terrific broths at 22°C and in the presence of 1% glucose. Protein expression was induced by adding 0.05 mM IPTG when the cells reach to 0.5 OD_600_. Cells were harvested 12 hours post-induction and analyzed by SDS-PAGE. The highest amount of biomass (~27.7 g L^-1^ culture) was obtained in Super broth followed by Terrific broth (~18.3 g L^-1^), and LB broth (16.7 g L^-1^). Growth in M9 minimal broth resulted in the lowest amount of biomass production (7.5 g L^-1^). Expression of CA was similar in LB, Super, and Terrific broth, and lowest in M9 minimal broth when equal amount of biomass subjected to SDS-PAGE analysis (Figure 5A). However, since largest biomass was produced in Super broth, it resulted into highest amount of HIV-1 CA produced from 1L culture (Figure 5B).

Addition of 2-3% ethanol in culture medium [23,24] and incubation at 42°C [25] leads to the overexpression of chaperons, which in turn results in improved folding and enhanced solubility of certain proteins. We tested these conditions to improve the solubility of CA but did not observe any effect (data not shown). When analyzed using Wilkinson-Harrison statistical solubility model [26], the HIV-1 CA-6His found to have a CV-CV’ value of 0.57 and 63.8 percent chance of insolubility when overexpressed in *E. coli*. In our case, percentage solubility based on the band density (density of soluble band divided by the density of the soluble plus insoluble bands) was 60.65% and 65.35% for NiCo21(DE3) and NiCo21(DE3)-pACYC-RIL respectively. Based on our results and Wilkinson-Harrison statistical solubility model, we anticipate that CA may not be expressed 100% in soluble state, unless it is expressed as fusion protein with solubility enhancing partner such as maltose binding protein (MBP), NusA etc.

### Purification of HIV-1 CA from pSA-Hp24-6His-transfected NiCo21(DE3) E. coli

**Figure 6:**
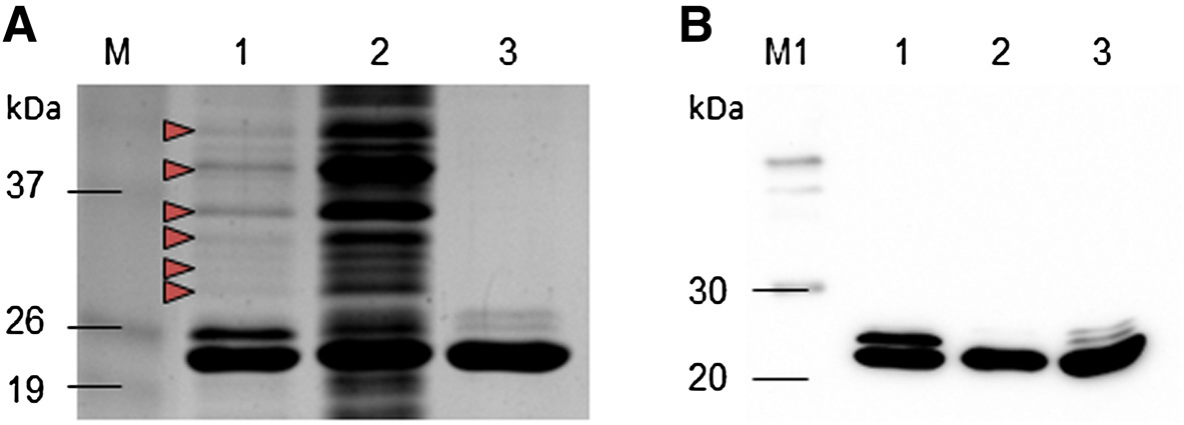
**Purification of HIV-1 CA expressed in NiCo21(DE3) *E. coli*.** NiCo21(DE3)-pACYC-RIL expressing HIV-1 CA were grown in presence of 0.05 mM IPTG at 22°C for 12 hours and processed to purify recombinant protein using Co^2+^ resin, chitin resin, and Chitin + Co^2+^ resins. Sample collected during the procedure were analyzed by SDS-PAGE **(A)** and western blot **(B)**. Lane M, BenchMark Pre-stained protein ladder; Lane M1, MagicMark XP Western Protein Standard; Lane 1, CA purified on Co^2+^ resin; Lane 2, CA purified on chitin resin; Lane 3, CA sequentially purified on chitin and Co^2+^ resins. Red arrowheads show contaminating proteins when CA was purified using Co^2+^ resin alone (Lane 1).

BL21(DE3) is a widely used *E. coli* strain for recombinant protein overexpression. Like other *E. coli* strains, BL21(DE3) contains a number of host proteins that are rich in nonconsecutive histidine residues. These histidine-rich proteins co-purify during IMAC procedures rendering recombinant protein preparations impure. The NiCo21(DE3) is a derivative of BL21(DE3) in which three histidine-rich proteins (*sly*D, *can*, *arn*A) are tagged with chitin binding domain and in one (*glm*S), the histidine-rich motifs are replaced with alanine [27]. Pre-adsorption of bacterial cell lysates on chitin beads results in the removal of major host histidine-rich proteins. Depleted fractions are then subjected to IMAC, which yields highly pure 6His-tagged recombinant protein preparations. We lysed the pSA-Hp24-6His-transformed NiCo21(DE3) and subjected the lysates to IMAC (Co^2+^ beads) alone, chitin beads alone, and chitin beads + IMAC purification. A considerable amount of contaminating proteins (shown by the arrowheads) co-purified with HIV- CA when purified using IMAC alone (Figure 6A, lane 1) or chitin beads alone (Figure 6A, lane 2). On the other hand, the HIV-1 CA was purified with un-noticeable (on Coomassie-stained gels) host protein contamination (Figure 6A, lane 3) when subjected to chitin beads + IMAC purification strategy. In some experiments, we first performed IMAC, and imidazole eluate was then treated with chitin beads to remove the host histidine-rich proteins. However, results were not as good, perhaps due to the presence of 150 mM imidazole in the eluate (results not shown).

**Figure 7:**
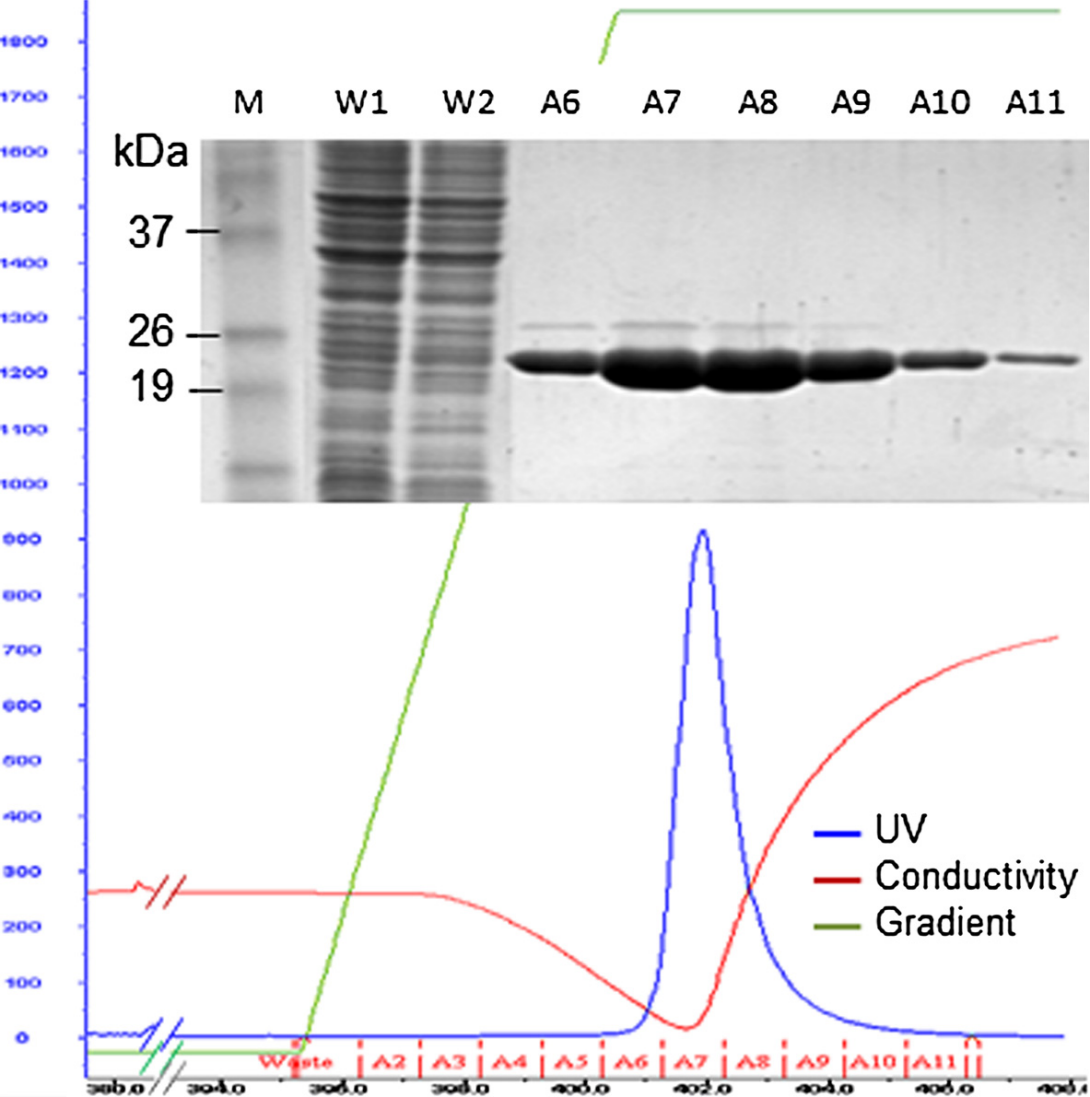
**FPLC and electrophoretic analysis of HIV-1 CA.** HIV-1 CA was purified using TALON® Superflow™ cobalt-based IMAC column on an FPLC system. SDS/PAGE (12%) analysis results (**inset**) of the eluted fractions as shown in the FPLC chromatogram. Fractions A6 to A11 were pooled as the high purity product. Lane M, BenchMark Pre-stained protein ladder; Lanes W1/W2, unbound protein in wash fractions; Lanes A6-A11, CA eluted in imidazole gradient.

To further demonstrate the purification efficiency, we treated the HIV-1 CA overexpressing NiCo21(DE3) lysates with chitin beads and subjected the chitin bead-treated lysates to TALON® Superflow™ cobalt-based IMAC column on FPLC system. The recombinant CA was eluted with a linear (5-150 mM) imidazole gradient. Elution of CA was monitored by absorbance at 280 nm. A representative FPLC chromatogram shows a single peak eluting at 150 mM imidazole, spanning A6-A11 fractions. We subjected these fractions to SDS-PAGE analysis, which shows a single 24 kDa band in all six fractions. The eluted protein in these fractions did not appear to be contaminated with other proteins (Figure 7).

Using the purification scheme described above, we yielded >6 mg of purified HIV-1 CA from 1g biomass of pSA-Hp24-6His-transformed NiCo21(DE3)/pACYC-RIL when propagated in super broth and induced with 0.05 mM IPTG for 12h at 21°C. The average yield and percentage recovery of recombinant HIV-1 CA from three independent 100 mL expression cultures is given in the Table 1.

**Table 1:** Yield and percentage recovery of recombinant HIV-1 CA

**Sample**	**Total protein (mg)**	**HIV CA (mg)**	**Purity (%)**
Soluble lysate	288	39	13.5
Chitin beads	166	25	15.06
IMAC	17.5	17	97.1

NiCo21(DE3)-pACYC-RIL expressing HIV-1 CA were grown in super broth the presence of 0.05 mM IPTG at 22°C for 12 hours and processed to purify recombinant protein as described in Methods. Quantities of recombinant HIV-1 CA are based on average values from three independent 100 mL expression cultures. HIV-1 CA was quantified by densitometry of bands on a western blots and the Qubit fluorometric total protein assay.

### Antigenicity of recombinant HIV-1 CA

**Figure 8:**
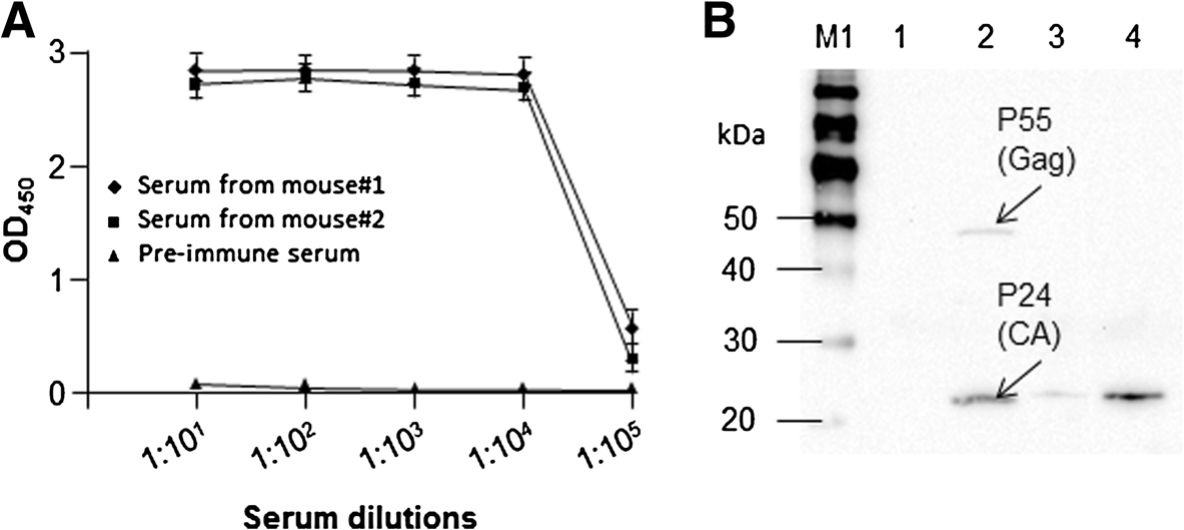
**Antigenicity of HIV-1 CA.** We immunized mice with purified CA as described in Methods. The sera from immunized mice were serially diluted and subjected to indirect ELISA. The pre-immune serum served as the negative control **(A)**. 293T cells were transfected with the pNL4-3 plasmid and cell lysates were analyzed by SDS-PAGE and Western blotting using serum from CA-immunized mice **(B)**. Lane M1, MagicMark XP Western Protein Standard; Lane 1, mock-transfected 293T cell lysate; Lane 2, pNL4.3-transfected 293T cell lysate; Lane 3, purified CA (10 ng); Lane 4, purified CA (100 ng).

This work is part of a project in which we are generating anti-CA antibody phage display library (manuscript in preparation). The ELISA plates coated with recombinant CA were used to determine the serum titer of immunized mice prior to harvesting anti-CA antibody mRNA from the spleens. Serum was diluted 1:10, 1:100, 1:1000, 1:10,000, and 1:100,000 respectively and assayed using indirect ELISA. Pre-immune serum was used as negative control. As shown in Figure 8A, the titer was higher than 1:100,000.

The antiserum was also used for immunoblot analysis of the cell lysates of pNL4.3-transfected/HIV-1 (NL4.3)-producing 293T cells and mock-transfected 293T cells (as negative control). Recombinant HIV-1 CA was used as the positive control. The antiserum specifically reacted with pNL4.3-transfected 293T cells and recombinant CA but not with the un-transfected 293T cells (Figure 8B). These results show that the recombinant CA had correct immunogenicity profile.

### Polymerization competency of HIV-1 CA

**Figure 9:**
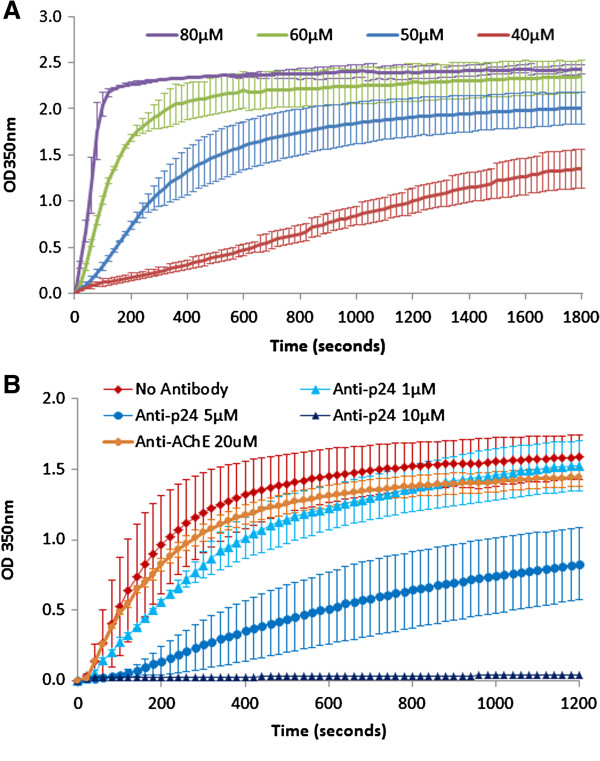
**Polymerization competency of HIV-1 CA.** Purified CA (40-80 μM) was mixed with 50 mM sodium phosphate buffer (pH 8.0) and the polymerization was induced by the addition of 2.0 M NaCl solution. Polymerization was monitored by measuring absorbance at 350 nm every 10 s for up to 60 min **(A)**. To further confirm that purified HIV-1 CA could be used for the identification of CA assembly inhibitors, we incubated 50 μM HIV-1 CA with varying concentrations (1,5,10 μM) of CA-specific anti-p24 MAb. Anti-Acetylcholinesterase antibody was used as a negative control **(B)**.

To show that purified CA was biologically active (capable of polymerizing), we subjected the recombinant protein to an in vitro polymerization assay. The recombinant CA was allowed to polymerize under high salt conditions and the change in turbidity (measured at 350 nm) over time (up to 60 min) was recorded. Kinetic traces of CA exhibited a typical sigmoidal time-dependent protein aggregation/polymerization curve for 80, 60 and 50 μM CA (Figure 9A). Curves obtained for 40 μM CA showed a lag phase (Figure 9A) that was due to the low concentration of CA and characteristic of a nucleation step. These results showed that purified CA was biologically active and suitable to study polymerization/inhibition of polymerization of the intact capsid protein (CA) of HIV-1 into mature capsid-like particles.

To further demonstrate that the purified HIV-CA could be used for the identification of CA assembly inhibitors, we incubated 50 μM HIV-1 CA with varying concentrations (1,5,10 μM) of CA-specific anti-p24 antibody. Incubation of HIV-CA with anti-p24 antibody resulted in a dose-dependent inhibition of CA polymerization. Incubation with 10 μM anti-p24 resulted in a complete inhibition of CA assembly, whereas 5 μM antibody delayed the rate of CA polymerization (Figure 9B). To rule out the possibility of non-specific inhibition of CA assembly due to the presence of large antibody molecules in the reaction mix, we incubated the HIV-1 CA with anti-AChE antibody. Presence of 20 μM (twice as much as anti-p24) anti-AChE had no effect on CA polymerization (Figure 9B). This suggests that CA polymerization inhibition in the presence of anti-p24 was due to the specific binding to the CA molecules.

## Discussion

Emergence of anti-retroviral (ARV) drug-resistant quasispecies of HIV is a major obstacle in the effective treatment of AIDS. To cope with the drug resistant mutants, there is a constant need of developing newer and better therapeutic regimens against novel targets. Due to its important role in both late and early stages of the HIV replication cycle, CA has attracted considerable attention as a potential target in recent years. Chemical compounds and peptides interfering with the capsid assembly have shown to alter the infectivity of the virions and prevented their release from the cells [2-6]. These studies typically need milligram quantities of highly pure and polymerization-competent CA. Due to the high cost of commercially available CA, it would by highly desirable to produce it in-house.

Bacterium *Escherichia coli* is by far the most robust and economical in vivo system for the production of heterologous recombinant proteins in scalable quantities. Unfortunately, HIV-1 CA expresses poorly in *E. coli* due to its use of rare codons. Codon optimization is one possibility but it can prove tedious and costly. Furthermore, codon optimization does not guarantee high yields of soluble protein since bacterial folding machinery can get saturated due to the high turnover rate of protein synthesis, and produced protein can end up in the inclusion bodies [22]. An alternative of codon optimization is to transform the expression host with plasmids expressing rare tRNA. We anticipated that the use of a host strain supplemented with rare tRNA should result in higher yields of recombinant CA. Indeed, when expressed in NiCo21(DE3) containing rare tRNA-expressing plasmids pACYC-RIL and/or pRARE2, there was a significant improvement in the yield of recombinant HIV-1 CA. Plasmid pACYC-RIL supplies tRNA for arginine (AGA, AGG), isoleucine (AUA), and leucine (CUA) whereas pRARE2 supplies tRNA for arginine (AGA, AGG, CGG), isoleucine (AUA), leucine (CUA), proline (CCC), and glycine (GGA). However, protein expression was indifferent between the bacteria carrying pACYC-RIL or pRARE2. The highest number of rare codons in *p*24 gene are for arginine (10 rare codons) followed by isoleucine (4 rare codons), leucine (3 rare codons), and proline (2 rare codons). It appears that tRNA supplementation for frequently used rare codons (arginine, isoleucine, and leucine) was sufficient to improve the protein yield.

Lab-scale purification of HIV-1 CA has also been hampered due to its poor solubility when overexpressed in *E. coli*. While it is possible to purify functional proteins from the inclusion bodies, it is a time consuming process and increases the cost of production. Attempts have been made to improve the solubility of HIV-1 CA by gene modification. For instance, it has been reported that solubility can be improved by introducing a Cysteine at 230 position, which makes a disulfide bond with Cys210 and promotes proper folding of recombinant CA [10]. It was also shown that the substitutions of certain amino acids had no effect on the immunogenicity of the recombinant protein [10]. However, whether these changes would have altered the polymerization competence of CA was not discussed. Since the recombinant CA produced in present study was intended to be used for generating/screening anti-CA phage display library and screening polymerization inhibiting single chain Fv antibodies, we used wild-type sequence. By systematically optimizing the culture conditions (IPTG concentration, temperature, culture medium composition), we managed to produce milligram quantities of recombinant protein in soluble state. It would be interesting to note that as little as 0.05 mM IPTG was sufficient to induce the recombinant protein expression. Use of less IPTG in the culture medium would also result in the economical production of recombinant protein. Even though the ratio of soluble *vs* insoluble protein was highest for cultures incubated at 18°C, the final yield was greatest when the cultures were incubated at room temperature (22 ±2°C). Room temperature incubation is also convenient and economical compared to 18°C, which needs refrigerated incubators.

IMAC is one of the most popular methods for the purification of poly-histidine-tagged recombinant proteins. Unfortunately, poly-histidine-tagged recombinant proteins that are isolated by IMAC are frequently contaminated with endogenous *E. coli* metal binding proteins. Some 17 *E. coli* IMAC contaminating proteins have been described, 15 of which elute from Ni-NTA at >55 mM imidazole. These contaminating proteins include CRP, Fur, ArgE, DnaK, SlyD, GlmS, GlgA, ODO1, ODO2, Can (YadF), ArnA (YfbG), AceE, GroES, and GroEL. Since His-tagged proteins are typically eluted between 60-150 mM imidazole, the IMAC contaminants co-elute rendering target protein preparations impure. Secondary chromatographic steps, e.g., size exclusion, protein specific (Heparin affinity), immunoaffinity, and dual affinity tag chromatography have been suggested. However, these additional procedures result in more operative time, additional cost, and lower overall yields.

To address this problem, Robichon et al. has described NiCo21(DE3) strain specifically engineered to minimize major *E. coli* protein contaminants (SlyD, GlmS, Can, ArnA) of IMAC fractions [27]. This strain expresses the endogenous proteins SlyD, Can, and ArnA fused at their C terminus to a chitin binding domain (CBD) and the protein GlmS, with six surface histidines replaced by alanines [27]. Desired recombinant protein(s) produced in NiCo21(DE3) are separated from CBD-tagged IMAC contaminating proteins by adsorbing them on chitin beads.

As shown in the results, purification of HIV-1 CA using IMAC alone resulted in CA contaminated with several host proteins. When the soluble lysate was pre-treated with chitin beads and then subjected to IMAC, highly pure CA was obtained. The only downside of this purification scheme is the employment of two successive purification steps, one using chitin beads, and second using IMAC. However, this scheme of purification is still better than those where additional chromatographic steps are employed. Chitin beads are cheap, can be regenerated multiple times, and above all, compatible with commonly used IMAC buffers. The loss of desired protein in chitin bead pre-treatment was minimal in our case. While in process of preparing this manuscript, Andersen et al. reported the use of LOBSTR, a derivative of *E. coli* BL21(DE3) that carries gnomically modified copies of *arn*A and *sly*D [28]. Proteins translated from these modified genes exhibited reduced affinities to IMAC, resulting in higher purity of overexpressed heterologous proteins. While only two of the four major metal binding proteins are modified for reduced affinity to IMAC, use of LOBSTR has shown to result in significantly improved purification and without the additional chitin resin adsorption step. This will both reduce the cost and time.

We showed that the recombinant HIV-1 CA produced in this work was biologically active in the polymerization assay. We also showed that the polymerization of HIV-1 CA could be inhibited following its incubation with CA-specific anti-p24 antibody. We incubated the CA with an unrelated anti-AChE antibody and demonstrated that CA polymerization was specifically inhibited by the anti-p24 antibody. These data indicate that CA polymerization assay could be used to screen CA polymerization-inhibiting antibodies. CA is an intracellular target and therefore off-limit to antibodies that normally do not penetrate into the cytoplasm. However, strategies are being devised to develop antibodies capable of crossing the plasma membrane and interacting with intracellular targets [29-31].

A recent report has described the purification of HIV-1 CA using its intrinsic tendency to polymerize and depolymerize. Isolation of protein using this technique did result in the purification of highly pure and polymerization competent CA [11]. While the technique is novel, it still involves several steps, from ammonium sulfate precipitation to polymerization/de-polymerization, multiple dialyses, and anion exchange chromatography. The route we took to purify CA is conventional but it involves fewer steps and is highly reproducible. Use of approach described here has resulted in as much protein yield as compared to the method reported by Hung et al. (>6 mg versus 5-7 mg from each one gram of bacterial biomass). By cultivating the bacteria in Super broth, twice as much capsid protein can be produced from 1 L of the culture.

## Conclusion

In the current paper, we report on a methodical analysis of HIV-1 CA expression in *E. coli* based on the combination of expression vector, *E. coli* strain selection, and analysis of cultivation conditions and fermentation broth. The simple expression and purification procedure described in this work resulted in the production of highly pure and biologically active HIV-1 CA in good yields for potential use in drug discovery, diagnostics, and in vaccine production.

## Competing interests

The authors declare that they have no competing interests.
